# GDR (Genome Database for Rosaceae): integrated web resources for Rosaceae genomics and genetics research

**DOI:** 10.1186/1471-2105-5-130

**Published:** 2004-09-09

**Authors:** Sook Jung, Christopher Jesudurai, Margaret Staton, Zhidian Du, Stephen Ficklin, Ilhyung Cho, Albert Abbott, Jeffrey Tomkins, Dorrie Main

**Affiliations:** 1 Department of Genetics and Biochemistry, Clemson University, Clemson, SC, 29634, USA; 2 Clemson University Genomics Institute, Clemson University, Clemson, SC, 29634, USA; 3 Department of Computer Sciences, Saginaw Valley State University, University Center, MI, 48710, USA

## Abstract

**Background:**

Peach is being developed as a model organism for Rosaceae, an economically important family that includes fruits and ornamental plants such as apple, pear, strawberry, cherry, almond and rose. The genomics and genetics data of peach can play a significant role in the gene discovery and the genetic understanding of related species. The effective utilization of these peach resources, however, requires the development of an integrated and centralized database with associated analysis tools.

**Description:**

The Genome Database for Rosaceae (GDR) is a curated and integrated web-based relational database. GDR contains comprehensive data of the genetically anchored peach physical map, an annotated peach EST database, Rosaceae maps and markers and all publicly available Rosaceae sequences. Annotations of ESTs include contig assembly, putative function, simple sequence repeats, and anchored position to the peach physical map where applicable. Our integrated map viewer provides graphical interface to the genetic, transcriptome and physical mapping information. ESTs, BACs and markers can be queried by various categories and the search result sites are linked to the integrated map viewer or to the WebFPC physical map sites. In addition to browsing and querying the database, users can compare their sequences with the annotated GDR sequences via a dedicated sequence similarity server running either the BLAST or FASTA algorithm. To demonstrate the utility of the integrated and fully annotated database and analysis tools, we describe a case study where we anchored Rosaceae sequences to the peach physical and genetic map by sequence similarity.

**Conclusions:**

The GDR has been initiated to meet the major deficiency in Rosaceae genomics and genetics research, namely a centralized web database and bioinformatics tools for data storage, analysis and exchange. GDR can be accessed at .

## Background

In the United States and temperate regions throughout the world, the family Rosaceae ranks third in economic importance. The most important fruit producing crops include apple (*Malus*), pear (*Pyrus*), raspberries/blackberries (*Rubus*), strawberries (*Fragaria*), and stone fruits (*Prunus*) such as peach/nectarine, apricot, plum, cherry and almond [1]. Additionally, Rosaceae contains a wide variety of ornamental plants including roses, flowering cherry, crabapple, quince and pear. Peach is being developed as a model species for Rosaceae because of its small genome size [haploid size of 300 Mb [3]], approximately twice that of *Arabidopsis *and other characteristics: a relatively short juvenile period (2–3 yrs) and extensive genetics and genomics resources such as molecular marker maps, interesting mutants and clone library resources [1]. In addition, it has been demonstrated that molecular marker tools developed in peach are easily applied to other species in the family [5-8]. Developing genetic resources for a model organism can greatly accelerate the genetic understanding of the individual member species in the same family. The utilization of rice physical mapping resources in the study of other crops within Poaceae is an excellent example of the usefulness of a comparative genomics approach [2]. Two major efforts to develop peach as a model for genomics of Rosaceae have been initiated: (1) Structural genomics – the development of a complete physical map of the peach genome and the anchoring of the genetic markers of many of the economically important Rosaceae species maps on this physical map (2) Functional genomics – the development of an extensive EST database for fruit, shoot and seed tissues and integration of the tentative unigene set onto the physical and genetic maps of peach.

The volume and the complexity of the data being produced by these peach genomics projects, in addition to the rapidly accumulating genomics and genetics data for other important rosaceous species, necessitate the development of a properly curated and integrated scientific database. Such a database will help scientists to efficiently access, analyze, integrate and apply the data to their own research in a timely manner. RoseDB, which focused on apple genetics and cherry genomics, has been decommissioned and now only exists as a mirror site at INRA. To meet the major need for a centralized and integrated web-database for genomics and genetics research in Rosaceae, the Genome Database for Rosaceae (GDR; ) has been initiated. The goals of the GDR are (1) to develop an organized and integrated web resource for peach genomics data to facilitate the gene discovery in other member species by a comparative mapping approach, (2) to collect and integrate all Rosaceae genomics data, (3) to develop online tools and resources for the Rosaceae community. In this paper, we describe the structure and content of the database, we review the database access utility and tools and we report a case study where we mapped publicly available Rosaceae sequences to the peach physical and genetic maps by sequence similarity.

## Construction and content

### GDR data

#### EST data source and annotation

An international cooperative project is in progress to develop an extensive peach EST database from a variety of vegetative and reproductive tissues of peach and almond. Currently, GDR contains 9984 ESTs from developing fruit of peach and 2794 ESTs from developing seed of almond. This translates into 3843 tentative unigenes for peach and 773 for almond.

The peach and almond ESTs are being processed at the Clemson University Genomics Institute (CUGI) utilizing publicly available software, integrated in a fully automated in-house developed script (CUGIEST). The processing occurs in three stages: trace file processing to identify a filtered, high quality clone library, assembly of high quality sequences to produce longer transcripts and reduce redundancy, and sequence annotation. Annotation consists of pairwise comparison of both the filtered clone library and the EST contig consensus sequences against the GenBank nr protein database using the fastx3.4 algorithm [9]. The ten most significant matches with the expectation values (EXP) less than 1 × e^-9^, for each contig and individual clone in the library are recorded. The unigene data set is then derived by selecting the clone that best represents each contig (the clone with the most significant EXP for the homology search) and the singletons that have either unique protein matches or no known significant matches. Peach ESTs were further annotated by Gene Ontology (GO) assignment based on the single "best hit" match against the SWISS-PROT database. Of the 1552 sequences from the putative peach unigene set that had matches with the SWISS-PROT database, 1439 could tentatively be assigned GO classifications. Additional sequence annotation includes computational analysis for simple sequence repeats (SSR) and open reading frame (ORF) on both the filtered clone library and the contig library. SSR analysis was preformed using a modified version (CUGISSR) of a Perl script SSRIT [10] with parameters set to detect di- to pentanucleotide with length greater than 18 bp. To examine the location of SSRs in the EST sequences in relation to the putative coding region, CUGISSR uses the FLIP [11] program which is available at the OGMP (Organelle Genome Megasequencing Project), Biochemistry department, University of Montreal . FLIP is a UNIX C program that finds/translates ORFs (open reading frames) in sequences. Using the FLIP output, CUGISSR selects the longest ORF as the putative coding region and reports the location of SSRs in relation to the putative coding region.

In addition to the peach and almond ESTs processed by CUGI, all the publicly available Rosaceae EST data are daily downloaded from GenBank dbEST and annotated with the top ten most significant matches (EXP < 1 × e^-9^) following a monthly homology search of GenBank nr protein database using the fastx34 algorithm.

#### Genetically anchored physical map and transcript map data

The genetically anchored physical map for peach is under development using peach BAC libraries [1]. It is being constructed using an approach that employs a combination of hybridization of mapped markers and BAC fingerprinting [12]. For BAC fingerprinting, FPC [13] is used for automatic assembly of the bands. Hybridization of mapped markers to BAC clones aids in the physical mapping process and also enables researchers to identify the BACs containing these markers. To date, over 250 genetic markers such as RFLPs, AFLPs and SSRs from several molecular genetic maps have been hybridized to BAC clones. Through this, the peach physical map has been anchored on the general *Prunus *map [5]. Additional 3,000 peach ESTs from the unigene set are currently being mapped to the peach physical map to develop a transcript map of peach fruit ESTs. The EST hybridization is being done as an international cooperative project to develop a functional genomic database for peach. The EST hybridization results are sent to the peach physical mapping team in Clemson University. The overall BAC fingerprinting results and EST/marker hybridization data, stored in an FPC output file and/or spread sheets, are submitted to GDR by the Clemson peach physical mapping team.

### Database and software design and implementation

The GDR is a relational database implemented using Oracle Database Management System version 9.2.0. Currently, the database is composed of 28 tables which store all the data for EST processing, assembly and annotation, SSR analysis, BAC clones, libraries, genetic markers and maps that are used for BAC hybridization, results of the hybridization of markers and ESTs to BAC clones, contact information, and publications.

EST data processing, annotation, and uploading of the database are fully automated using a series of scripts written in Perl version 5.8.2. Daily download, annotation and upload of GenBank Rosaceae ESTs are also automatically performed by a series of Perl scripts. Data for BAC hybridization, genetic maps and markers which are submitted from researchers are examined by a curator for any potential errors and then uploaded using Perl scripts. BAC contig data for the developing peach physical map are uploaded directly from the FPC output file to our oracle database using Perl scripts.

Web interfaces for database query and the query result pages are mostly developed using Java Server Page (JSP). JSPs are more efficient, easier to use, more powerful and more portable than traditional CGI and many alternative CGI [4]. BAC contigs of the developing peach physical maps are displayed using WebFPC and WebChrom which are downloaded from an Arizona Institute of Genomics web site . We have developed a map viewer to provide users with a convenient access to the integrated genetic, physical and transcript map information. Our map viewer is developed using Scalable Vector Graphics (SVG). The SVG viewer plug-in can be freely downloaded from the Adobe Website  and the system requirements can be found at their website . Our map viewer program accesses our underlying relational database to dynamically generate an integrated genetic and transcript map with a direct link to the WebFPC physical map from each marker.

## Utility and discussion

### Database access and tools

The GDR website is composed of general information pages, database query/browse interfaces and other tools such as map viewer and sequence similarity server. The GDR web pages are extensively linked such that users can easily access the data of interest regardless of the navigation starting point. For example, the EST detail pages have links to the BAC detail pages, marker detail pages or map viewer for the ESTs that are anchored to BACs, markers or maps. Similarly, the BAC detail pages have links to EST detail page, marker detail page, WebFPC and map viewer. Users can also access the data detail pages from the map viewer or WebFPC/WebChrom. Instead of displaying the entire EST or BAC data in one page, we used the right hand side navigation bars to help users find specific information easily and quickly. A general GDR navigation tool bar is also included in each page to help provide a more user-friendly interface.

#### Database search interface

The generic search site allows users to select data types such as EST, BAC and Marker, and search by name. Users can also follow the link to perform more detailed search for each data type. In the EST search site, users can search either the CUGI peach EST database or Rosaceae EST database downloaded from GenBank. ESTs can be searched by their name(s) and annotation features such as whether the EST belongs to a contig, is a unigene, is used as a probe and has SSRs, or any combination thereof. The EST details page, instead of displaying all the details in one page, initially displays the clone information and the sequence with a side bar containing links to library detail, assembly/unigene information, sequence homology, SSR information, Map position and anchored BACs. Each page linked from this side bar has the same side bar for easy navigation between the features. The Sequence homology page shows the most significant matches (EXP < 1e-9) in the Genbank nr protein database from the fastx sequence similarity search. The SSR information page shows the sequence along with the computationally derived SSRs to help users in the primer design for SSR marker development. The longest putative open reading frame (ORF) is also marked in color in the sequence along with the SSRs. SSRs in the non-coding region tend to be more polymorphic and those in the coding region tend to be more transferable among species, so the information of SSR position in a gene structure will be useful for marker development. The Map position page allows users to view the ESTs' map position using our Map Viewer. Users can retrieve the anchored BAC clones for the EST of interest and all the other ESTs and markers that hybridized to the same BAC through the anchored BACs page. The assembly/unigene information page displays the assembly results which include the contig name and the unigene clone that best represents the contig. The contig name is linked to a contig page that displays the contig sequence with a side bar containing links to the comprising ESTs, sequence homology and SSR information. As ESTs with no match to the GenBank proteins or with no SSRs can still assemble into contigs with a match or SSRs, users may get further annotation results of their ESTs of interest by visiting the contig detail site. In addition, contigs may have longer sequences surrounding the SSRs, allowing more flexibility in the primer design for marker development.

In the BAC search site, users can search BACs by name or by probe specifications used for BAC hybridization. The search results site and the linked sites provide users with all the data about the BAC, such as the BAC contigs that the BAC belongs to, other BACs in the contig, probes that hybridized to the BACs, the detailed data about the probes, and link to the WebFPC physical map. Markers can be searched by name or features such as map name, type, and source organism. Similarly with the BAC search result sites, the maker search results sites leads users to pages with the marker information, anchored BACs, other markers and ESTs hybridized to the same BACs and link to the Map Viewer and the WebFPC physical map.

#### Graphical interface to maps

**Figure 1:**
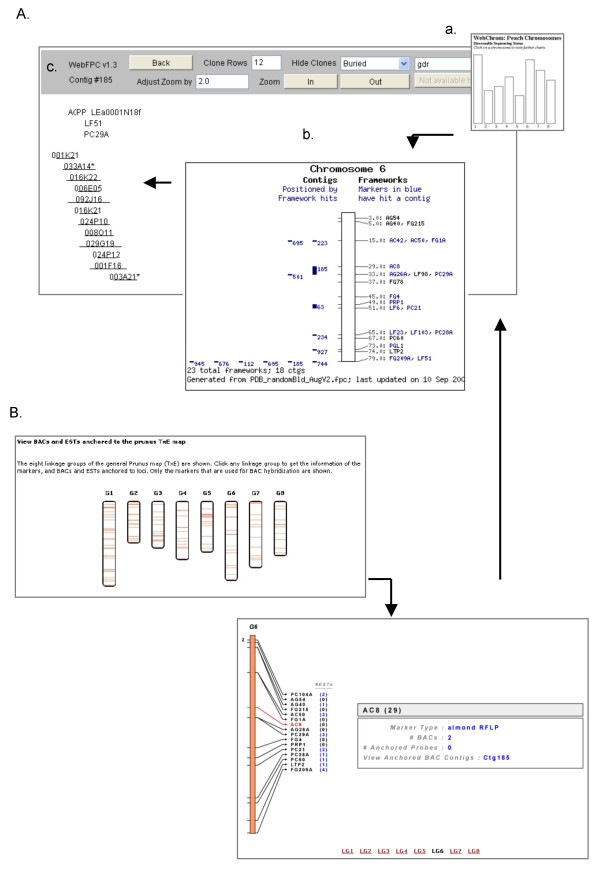
Graphical interface to maps. A. Peach WebChrom and peach WebFPC: a. the opening page of peach WebChrom with eight linkage group of the general *prunus *map; b. an example of individual linkage group pages (linkage group 6) with the associated markers and the contigs; c. an example of the directly linked FPC page with the developing BAC contigs and anchored probes (contig #185). B. Map Viewer: Map viewer displays the general *Prunus *map with anchored ESTs and direct link to WebFPC.

GDR hosts peach WebFPC and WebChrom to allow users to view the developing peach FPC contigs. Peach WebChrom displays the eight linkage groups of the general *Prunus *map and each linkage group has a link to a page where the developing contigs are located by the linkage group. Each contig has a direct link to WebFPC in which the individual BAC clones are displayed (Figure 1A). GDR also provides a graphical tool for users to access the integrated genetic, transcript and physical map information. The map viewer displays the general *Prunus *map [5] with the number of ESTs anchored to each locus (Figure 1B). The EST details page is linked from the map so that users can get all the data about the ESTs that are anchored to the loci of interest. When a locus is selected, a box appears to display marker type, the number of anchored BACs and the number of other probes that share the same BACs. Each number entry has links to a page where the detailed information is displayed. Also shown in the box are BAC contig names that are anchored to the loci. The BAC contig names have links to WebFPC so that users can directly access the physical map via this route (Figure 1B).

#### Sequence similarity server

GDR also has a BLAST and FASTA sequence similarity search server that allows users to conduct homology searches between their sequences of interest and the various sequence data sets including annotated sequences in GDR. Users can select the database (e.g. peach ESTs, peach unigenes, mapped peach unigenes, GenBank Rosaceae ESTs, GenBank Rosaceae proteins etc) and various search parameters. Users can upload batch sequence files and the parsed results from the search are formatted in a spread sheet and sent to users by email. When the query sequence has a match to the annotated GDR sequences, users can retrieve all the information such as putative function, SSRs, and the anchored map positions via a hyperlink in the excel spreadsheet. Our sequence similarity server, specifically designed for Rosaceae researchers, will help users utilize the developing peach resources in the studies of other Rosaceae species. For example, as described in the case study below, sequences derived from other Rosaceae species could be immediately anchored to the various Rosaceae maps and the physical map when the query sequences show significant similarity to the mapped peach ESTs.

### Case study: Mapping of Rosaceae sequences onto Rosaceae maps by sequence similarity

**Figure 2:**
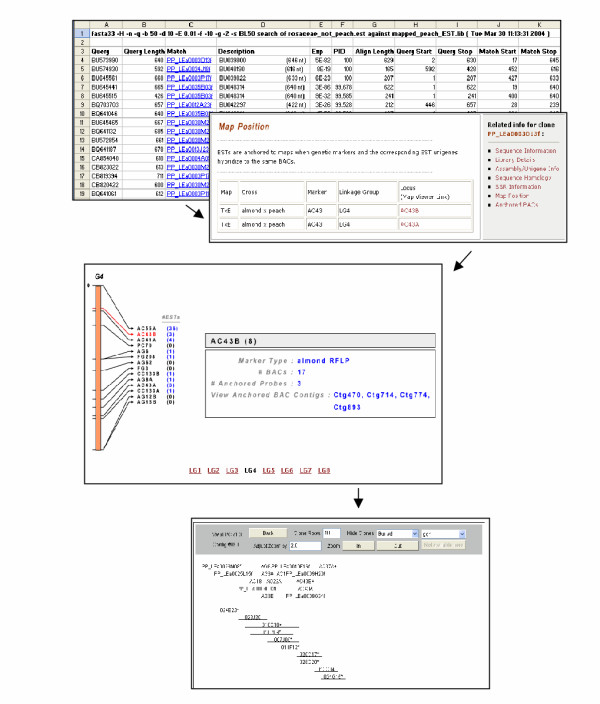
Case Study: Hyperlink to map viewers from the sequence similarity search result. This figure shows snapshots of hyperlinked pages to illustrate that users can obtain putative genetic and physical map positions of the query sequences when the sequences have match to mapped peach ESTs. A. A spread sheet with sequence similarity search results B. EST detail page with map position C. Map Viewer D. WebFPC.

**Figure 3:**
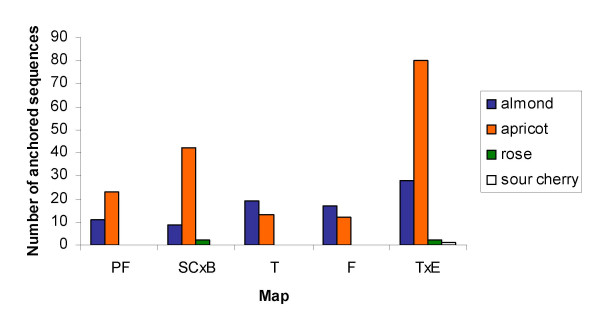
Case Study result: Number of anchored Rosaceae sequences to various Rosaceae genetic maps T × E: 'Texas' × 'Earlygold'; F or T: F × T ('Ferragnes' × 'Tuono'); P × F: ('IF310828' × P. ferganensis) × 'IF310828'; SC × B: 'Suncrest' × 'Bailey'

We report here a case study illustrating the utility of our sequence similarity server and other integrated GDR web resources. In this study, we performed a sequence similarity search using the FASTA algorithm with the non-peach Rosaceae sequences against the mapped peach ESTs to annotate Rosaceae sequences with map positions. A fasta formatted file with a total of 16258 publicly available non peach Rosaceae sequences was uploaded to the GDR FASTA server. We selected the mapped peach database and used the default parameters. The search results returned from the server are formatted in a spread sheet for easy browsing and the match names are hyperlinked to both GenBank and the GDR web site (Figure 2). By following the GDR link, users can get the anchored position in the genetic and physical maps as well as other annotation results such as putative function (Figure 2). To summarize our results, we used 259 query/match pairs which have a percent identity over 95 and an align-length greater than 100 nucleotides. The majority of the query sequences that showed high similarity to peach ESTs were *Prunus *sequences such as apricot (*Prunus armeniaca*), almond (*Prunus dulcis*) and sour cherry (*Prunus cerasus*). This was expected as peach also belongs to the genera *Prunus*. The 259 query/match pairs consisted of 209 Rosaceae sequences and 61 mapped peach ESTs. The matching of multiple Rosaceae sequences to single peach sequences was expected since the Rosaceae sequences were not assembled and therefore potentially contains multiple sequences representing the same gene. The 209 Rosaceae sequences were anchored to 38 different loci in four different Rosaceae maps. The number of sequences from each Rosaceae species that anchored to each map is shown in Figure 3. This study demonstrates the usefulness of applying a comparative genomics approach to Rosaceae genomics using the GDR as a data mining tool. The entire data for the mapped Rosaceae sequences with anchored map positions are available at .

### Future development

We plan to incorporate more Rosaceae genomics and genetics data from researchers worldwide as well as data from the ongoing *Prunus *genomics projects. Data to be added in the near future include apple ESTs, strawberry ESTs, rose ESTs and apricot map/marker data from collaborators. When the genomics projects from *Prunus *are finished, we will host 10–15,000 unique ESTs from a variety of vegetative and reproductive tissues of peach and almond, the complete peach physical map with anchored genetic markers and unique ESTs. In addition to adding new data, future development efforts will focus on improving the tools and functionality of the web interface such as an advanced search site with options for search/display categories, full sequence processing facilities for Rosaceae researchers, a newsgroup for the Rosaceae community, a site for Rosaceae literature, and more analysis tools such as an interactive contig viewer and a comparative map viewer.

## Conclusions

The GDR is initiated to support the genomics and genetics research in Rosaceae, which contains numerous economically important fruit trees and horticultural plants. Currently GDR contains all the genomics data for the Rosaceae model peach, maps and markers of Rosaceae species and all the publicly available Rosaceae ESTs. Our integrated database provides users with easy access and retrieval of the annotated data, and the web tools enable them to further analyze their data. With future plans, including more data acquisition and tool developments, GDR will play an important role in the timely and efficient analysis of the data, the exchange of results and ideas among researchers worldwide, the support of Rosaceae labs worldwide with Bioinformatics tools and the utilization of the data from the model species in the study of other Rosaceae species. The methodology and tools applied to develop GDR should be easily applied to develop other comparative genomic databases for different families.

## Availability

The GDR is publicly available and can be accessed at .

## List of abbreviations used

AFLP: Amplified Fragment Length Polymorphism

BAC: Bacterial Artificial Chromosome

CGI: Common Gateway Interface

EST: Expressed Sequence Tag

EXP: EXpectation Value

HTML: HyperText Markup Language

HTTP: HyperText Transfer Protocol

INRA: Institut National de la Recherche Agronomique

RFLP: Restriction Fragment Length Polymorphism

SSR: Simple Sequence Repeat

XML: eXtensible Markup Language

## Authors' contributions

SJ performed web interface design and programming for html pages and JSP search pages, participated in the database construction, carried out database curation, designed the Map Viewer and performed the case study. CJ wrote scripts for database upload and sequence processing, programmed Map Viewer and implemented WebFPC and WebChrom. MS participated in the database construction and wrote scripts for data upload and search pages for Genbank Rosaceae sequences. SF performs database administration. IC developed the application prototype for servlet-database connection and participated in the database construction. AA conceived of the project and participated in its design and coordination. ZD developed the sequence similarity server application. JT participated in the coordination of the project. DM conceived and supervised the project, participated in the database construction, carried out EST analysis, and provided input in the web interface design. All authors read and approved the final manuscript.
